# Supplementary material for: Multi-factorial examination of amplicon sequencing workflows from sample preparation to bioinformatic analysis
Source: BMC Microbiol. 2023 Apr 19;23:107. doi: 10.1186/s12866-023-02851-8 (PMC10114302; doi:10.1186/s12866-023-02851-8)
Supplement: Supplementary file 2 — Additional file 2. [file 12866_2023_2851_MOESM2_ESM.pdf]

Supplementary Material for "Multi-factorial examination of amplicon sequencing workflows from sample preparation to bioinformatic analysis" by Travis De Wolfe and Erik Wright.

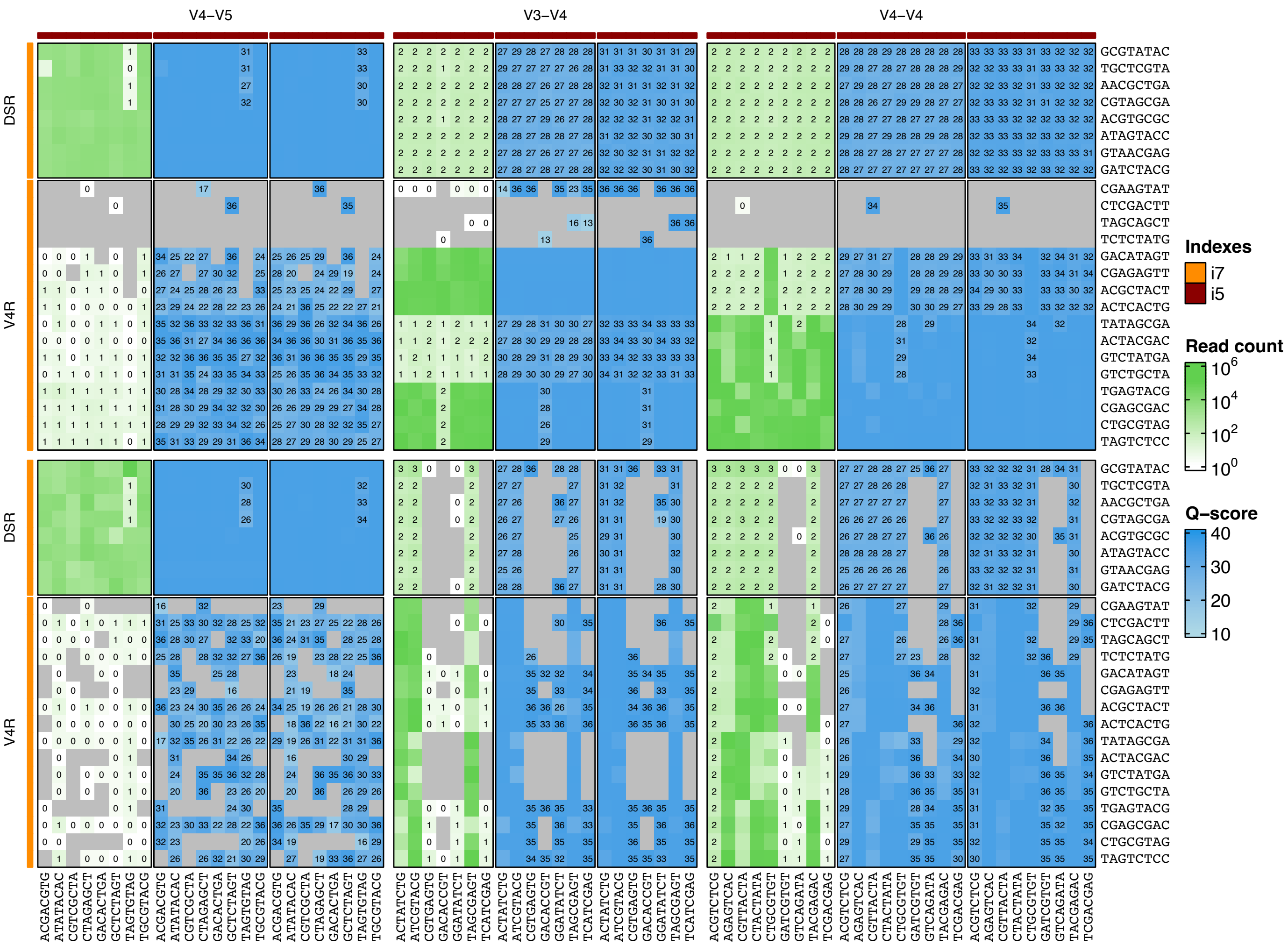

Supplemental figure 1. Index read count and Q-scores for expected or unexpected index combinations found among raw sequences. Heatmap cells containing a read count or Q-score for the respective index combination indicates the combination was not included as a sample in the sequencing run (unexpected, false positive). Grey heatmap cells indicate the index combination was not included as a sample in the sequencing run and it was not found in the resulting sequence data (true negative).

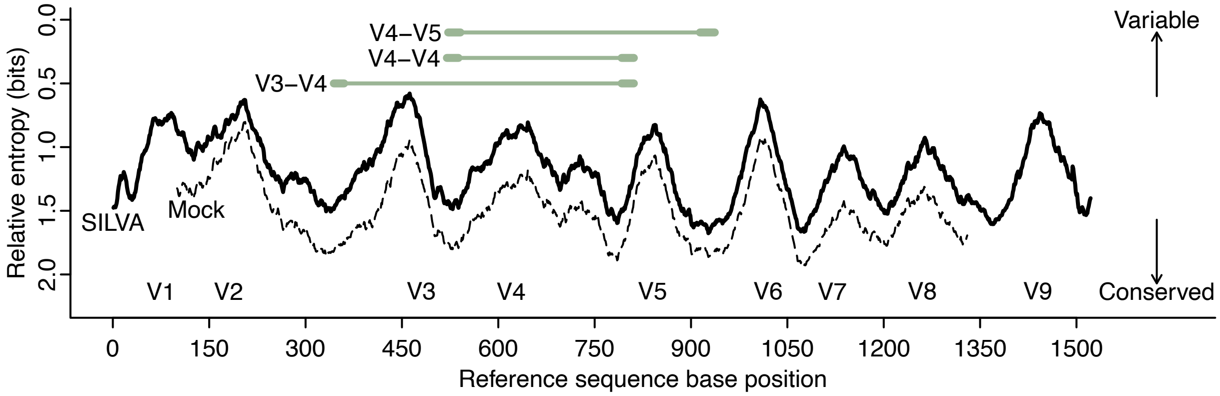

Supplemental figure 2. Entropy along the 16S rRNA reveals variable regions (V1 to V9) for inferring phylogenetic relationships. The primer set used in a 16S rRNA survey defines the variable region(s) that will be amplified during the PCR step of library preparation. Relative entropy values were determined from the SILVA r138 database and the mock bacterial community members used in this study. Nucleotide positions are numbered according to the *Escherichia coli* K-12 MG1655 16S rRNA positive-strand (GenBank accession number U00096). Note that the y-axis is reversed so variable regions display 'high' entropy on the plot.

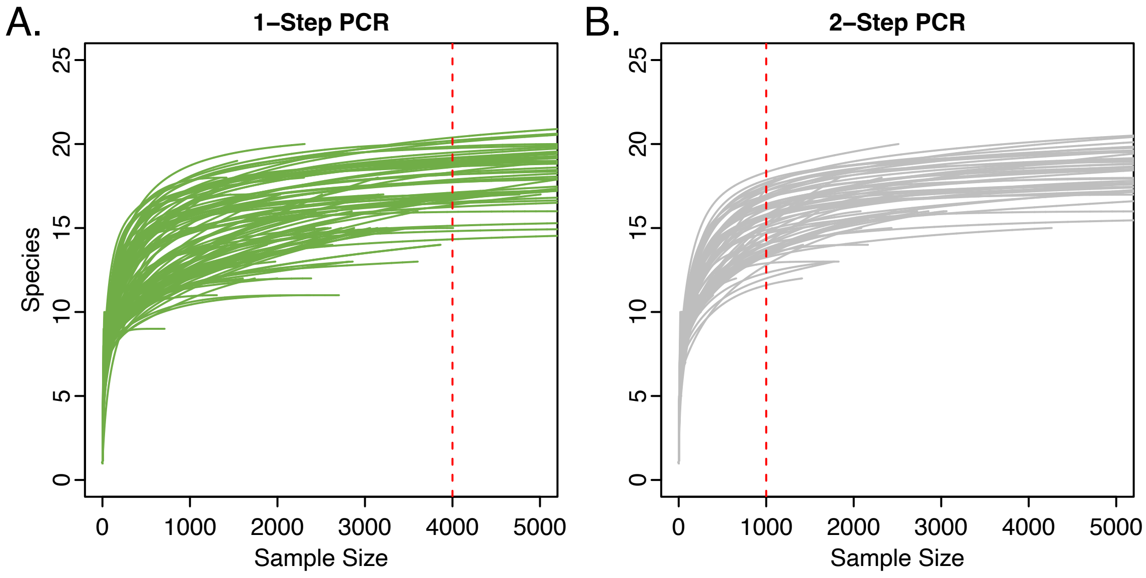

Supplemental figure 3. Evaluation of sequencing depth among sequencing runs included in this study. (A) Sub-sampling 4000 raw reads appeared sufficient to ensure that accuracy and coverage among samples indexed with 1-step PCR in our study are not due to chance. (B) Sub-sampling 1000 raw reads appeared moderately sufficient to ensure that accuracy and coverage among samples indexed with 2-step PCR in our study are not due to chance.

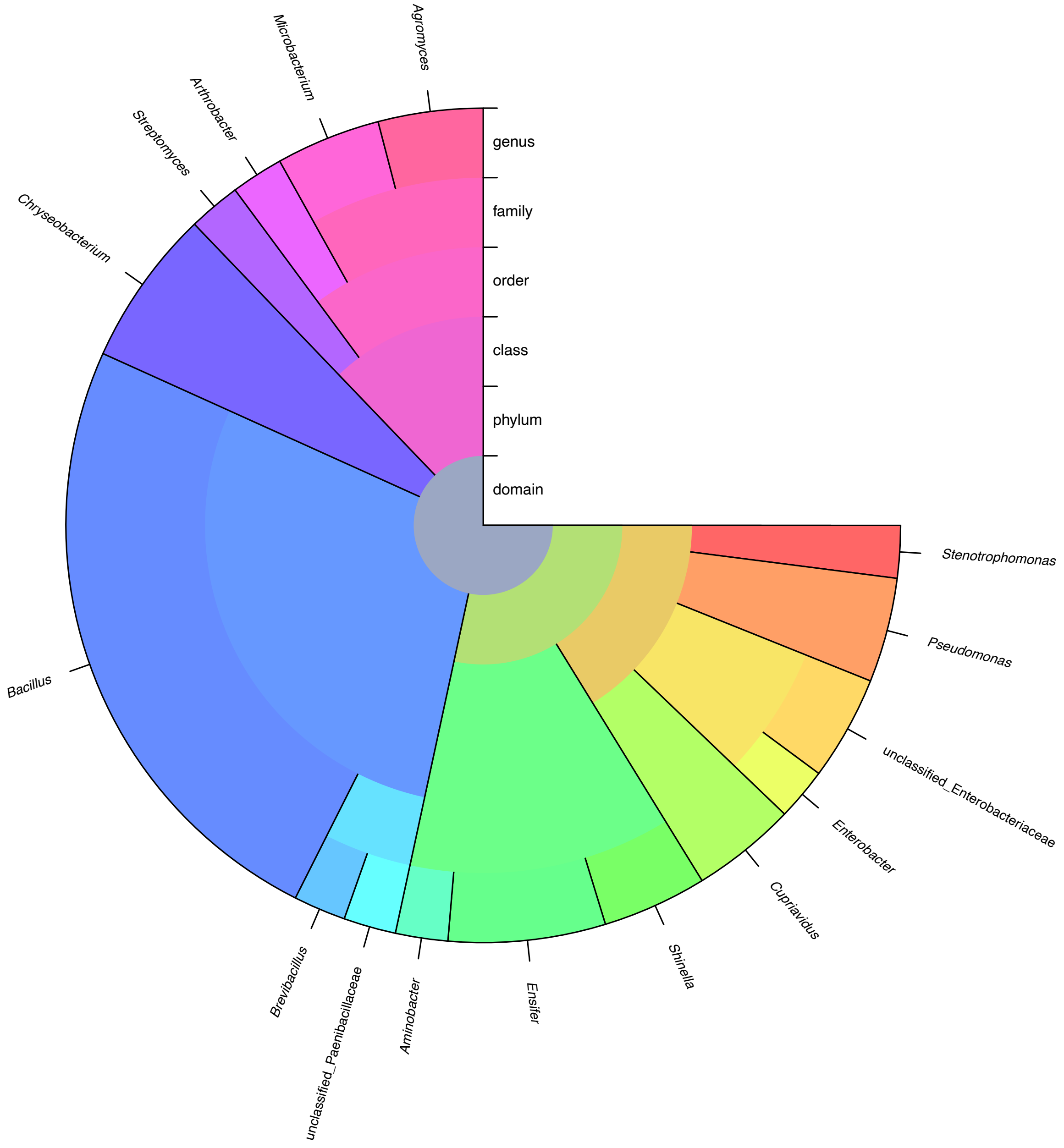

Supplemental figure 4. A total of 37 unique bacterial isolates were cultivated from soil and sequenced to construct a phylogenetically and taxonomically diverse mock community. Taxonomy was assigned by IDTAXA with the SILVA r138 database.

Supplemental table 1. Mock community composition

GeneBank	ID	Phylum	Class	Order	Family	Genus
OK033102	U001	Firmicutes	Bacilli	Bacillales	*Bacillaceae*	*Bacillus*
OK033103	U002	Actinobacteriota	Actinobacteria	Micrococcales	*Microbacteriaceae*	*Microbacterium*
MN186622	U004	Proteobacteria	Alphaproteobacteria	Rhizobiales	*Rhizobiaceae*	*Aminobacter*
MN186623	U005	Proteobacteria	Gammaproteobacteria	Xanthomonadales	*Xanthomonadaceae*	*Stenotrophomonas*
MN186625	U008	Proteobacteria	Gammaproteobacteria	Burkholderiales	*Burkholderiaceae*	*Cupriavidus*
MN186626	U009	Proteobacteria	Alphaproteobacteria	Rhizobiales	*Rhizobiaceae*	*Shinella*
MN186628	U011	Proteobacteria	Gammaproteobacteria	Enterobacterales	*Enterobacteriaceae*	*Enterobacter*
MN186629	U012	Proteobacteria	Gammaproteobacteria	Pseudomonadales	*Pseudomonadaceae*	*Pseudomonas*
MN186630	U013	Proteobacteria	Gammaproteobacteria	Enterobacterales	*Enterobacteriaceae*	Unclassified *Enterobacteriaceae*
MN186631	U015	Bacteroidota	Bacteroidia	Flavobacteriales	*Weeksellaceae*	*Chryseobacterium*
MN186632	U016	Actinobacteriota	Actinobacteria	Micrococcales	*Micrococcaceae*	*Arthrobacter*
MN186633	U017	Proteobacteria	Gammaproteobacteria	Burkholderiales	*Burkholderiaceae*	*Cupriavidus*
MN186634	U018	Proteobacteria	Alphaproteobacteria	Rhizobiales	*Rhizobiaceae*	*Shinella*
MN186637	U021	Firmicutes	Bacilli	Brevibacillales	*Brevibacillaceae*	*Brevibacillus*
MN186639	U023	Proteobacteria	Gammaproteobacteria	Pseudomonadales	*Pseudomonadaceae*	*Pseudomonas*
MN186641	U025	Bacteroidota	Bacteroidia	Flavobacteriales	*Weeksellaceae*	*Chryseobacterium*
MN186642	U027	Actinobacteriota	Actinobacteria	Micrococcales	*Microbacteriaceae*	*Agromyces*
MN186645	U031	Actinobacteriota	Actinobacteria	Streptomycetales	*Streptomycetaceae*	*Streptomyces*
MN186646	U032	Proteobacteria	Alphaproteobacteria	Rhizobiales	*Rhizobiaceae*	*Ensifer*
MN186647	U033	Actinobacteriota	Actinobacteria	Micrococcales	*Microbacteriaceae*	*Microbacterium*
MN186648	U034	Proteobacteria	Alphaproteobacteria	Rhizobiales	*Rhizobiaceae*	*Ensifer*
MN186649	U035	Firmicutes	Bacilli	Paenibacillales	*Paenibacillaceae*	Unclassified *Paenibacillaceae*
MN186654	U040	Firmicutes	Bacilli	Bacillales	*Bacillaceae*	*Bacillus*
MN186655	U041	Firmicutes	Bacilli	Bacillales	*Bacillaceae*	*Bacillus*

Taxonomy was assigned by IDTAXA with the SILVA r138 database.

Supplemental table 2. BLAST hits for potential off-target amplicon

Hit accession number	Percent identity	E-value	Bit score	Description	Off-target amplicon*	Read count
LN591134.1	79.03	0.066	50.9	*Cyprinus carpio*	Y	49
LN591134.1	79.03	0.066	50.9	*Cyprinus carpio*	Y	28
KR849800.1	100.00	5.73E-123	452.0	Uncultured bacterium	N	1

*Inexact matches that could not be classified with IDTAXA were evaluated for sequence similarity using BLAST v.2.10.0+. Sequence hits not matching to 16S rRNA were classified as off-target amplification

Supplemental table 3. Methodological factors evaluated in the present study

Indexing approaches	16S rRNA regions	Polymerases	Elongation times	Annealing temperatures	Program
1-step PCR	V3-V4	iTaq (Bio-Rad; 1725121)	15	T_m	DADA2
2-step PCR	V4-V4	SsoAdvanced (Bio-Rad; 1725270)	30	5 °C below T_m	QIIME2
	V4-V5	KAPA HiFi (Kapa Biosystems; KK2702)	60		mothur
			120		
			180		

Supplemental table 4. Primer and polymerase-wise annealing temperatures used in this study

Primer	Polymerase	Indexing approach	Annealing temperature	Annealing temperature (°C)
V3-V4	iTaq	1-step PCR	5 °C below T_m	53
V3-V4	SsoAdvanced	1-step PCR	5 °C below T_m	53
V3-V4	KAPA	1-step PCR	5 °C below T_m	62
V3-V4	iTaq	1-step PCR	T_m	58
V3-V4	SsoAdvanced	1-step PCR	T_m	58
V3-V4	KAPA	1-step PCR	T_m	67
V4-V4	iTaq	1-step PCR	5 °C below T_m	53
V4-V4	SsoAdvanced	1-step PCR	5 °C below T_m	53
V4-V4	KAPA	1-step PCR	5 °C below T_m	62
V4-V4	iTaq	1-step PCR	T_m	58
V4-V4	SsoAdvanced	1-step PCR	T_m	58
V4-V4	KAPA	1-step PCR	T_m	67
V4-V5	iTaq	1-step PCR	5 °C below T_m	53
V4-V5	SsoAdvanced	1-step PCR	5 °C below T_m	53
V4-V5	KAPA	1-step PCR	5 °C below T_m	62
V4-V5	iTaq	1-step PCR	T_m	58
V4-V5	SsoAdvanced	1-step PCR	T_m	58
V4-V5	KAPA	1-step PCR	T_m	67
V3-V4	iTaq	2-step PCR	5 °C below T_m	53
V3-V4	SsoAdvanced	2-step PCR	5 °C below T_m	53
V3-V4	KAPA	2-step PCR	5 °C below T_m	62
V3-V4	iTaq	2-step PCR	T_m	58
V3-V4	SsoAdvanced	2-step PCR	T_m	58
V3-V4	KAPA	2-step PCR	T_m	67
V4-V4	iTaq	2-step PCR	5 °C below T_m	53
V4-V4	SsoAdvanced	2-step PCR	5 °C below T_m	53
V4-V4	KAPA	2-step PCR	5 °C below T_m	62
V4-V4	iTaq	2-step PCR	T_m	58
V4-V4	SsoAdvanced	2-step PCR	T_m	58
V4-V4	KAPA	2-step PCR	T_m	67
V4-V5	iTaq	2-step PCR	5 °C below T_m	53
V4-V5	SsoAdvanced	2-step PCR	5 °C below T_m	53
V4-V5	KAPA	2-step PCR	5 °C below T_m	62
V4-V5	iTaq	2-step PCR	T_m	58
V4-V5	SsoAdvanced	2-step PCR	T_m	58
V4-V5	KAPA	2-step PCR	T_m	67

Supplemental table 5. Custom sequencing primers

Name	Sequence
V3_R1	TATGGTAATTGGCCTACGGGAGGCAGCAG
V4_R2	AGTCAGTCAGCCGGACTACNVGGGTWTCTAAT
V34_V4_index	ATTAGAWACCCBNGTAGTCCGGCTGACTGACT
V4_R1	TATGGTAATTGTGTGYCAGCMGCCGCGGTAA
V45_R1	TATGGTAATTAAGYCAGCMGCMGCGGTAATAC
V45_R2	AGTCAGTCAGAAGYCCCCGTCWATTCMTTTGAGTTT
V45_index	AAACTCAAAKGAATWGACGGGGRCTTCTGACTGACT
